# Comparison of On-Site Versus Remote Mobile Device Support in the Framingham Heart Study Using the Health eHeart Study for Digital Follow-up: Randomized Pilot Study Set Within an Observational Study Design

**DOI:** 10.2196/13238

**Published:** 2019-09-30

**Authors:** Nicole L Spartano, Honghuang Lin, Fangui Sun, Kathryn L Lunetta, Ludovic Trinquart, Maureen Valentino, Emily S Manders, Mark J Pletcher, Gregory M Marcus, David D McManus, Emelia J Benjamin, Caroline S Fox, Jeffrey E Olgin, Joanne M Murabito

**Affiliations:** 1 Section of Endocrinology, Diabetes, Nutrition, and Weight Management Boston University School of Medicine Boston, MA United States; 2 Framingham Heart Study Framingham, MA United States; 3 Section of Computational Biomedicine Department of Medicine Boston University School of Medicine Boston, MA United States; 4 Department of Biostatistics Boston University School of Public Health Boston, MA United States; 5 Department of Epidemiology and Biostatistics University of California, San Francisco San Francisco, CA United States; 6 Division of Hospital Medicine University of California, San Francisco San Francisco, CA United States; 7 Department of Medicine University of Massachusetts Medical School Worcester, MA United States; 8 Boston University School of Medicine Boston, MA United States; 9 Department of Epidemiology Boston University School of Public Health Boston, MA United States; 10 Merck Research Laboratories Boston, MA United States; 11 Section of General Internal Medicine Department of Medicine Boston University School of Medicine Boston, MA United States

**Keywords:** wearable electronic devices, cell phone, fitness trackers, electrocardiography, epidemiology

## Abstract

**Background:**

New electronic cohort (e-Cohort) study designs provide resource-effective methods for collecting participant data. It is unclear if implementing an e-Cohort study without direct, in-person participant contact can achieve successful participation rates.

**Objective:**

The objective of this study was to compare 2 distinct enrollment methods for setting up mobile health (mHealth) devices and to assess the ongoing adherence to device use in an e-Cohort pilot study.

**Methods:**

We coenrolled participants from the Framingham Heart Study (FHS) into the FHS–Health eHeart (HeH) pilot study, a digital cohort with infrastructure for collecting mHealth data. FHS participants who had an email address and smartphone were randomized to our FHS-HeH pilot study into 1 of 2 study arms: remote versus on-site support. We oversampled older adults (age ≥65 years), with a target of enrolling 20% of our sample as older adults. In the remote arm, participants received an email containing a link to enrollment website and, upon enrollment, were sent 4 smartphone-connectable sensor devices. Participants in the on-site arm were invited to visit an in-person FHS facility and were provided in-person support for enrollment and connecting the devices. Device data were tracked for at least 5 months.

**Results:**

Compared with the individuals who declined, individuals who consented to our pilot study (on-site, n=101; remote, n=93) were more likely to be women, highly educated, and younger. In the on-site arm, the connection and initial use of devices was ≥20% higher than the remote arm (mean percent difference was 25% [95% CI 17-35] for activity monitor, 22% [95% CI 12-32] for blood pressure cuff, 20% [95% CI 10-30] for scale, and 43% [95% CI 30-55] for electrocardiogram), with device connection rates in the on-site arm of 99%, 95%, 95%, and 84%. Once connected, continued device use over the 5-month study period was similar between the study arms.

**Conclusions:**

Our pilot study demonstrated that the deployment of mobile devices among middle-aged and older adults in the context of an on-site clinic visit was associated with higher initial rates of device use as compared with offering only remote support. Once connected, the device use was similar in both groups.

## Introduction

### Background

Recent advances in mobile health (mHealth) technology have improved the feasibility of collecting digital data and have the potential to revolutionize both research and health care delivery [1-4]. The term mHealth technology refers to the use of smartphones and other mobile devices for personal health monitoring, health care delivery, or research [5]. Expert recommendations from the National Institutes of Health (NIH) National Heart, Lung, and Blood Institute (NHLBI) advocated for using innovative approaches, such as study designs that utilize mHealth technology, to provide new opportunities for population science [6]. Innovative electronic cohort (*e-Cohort*) study designs that incorporate mHealth technology into traditional cohort studies have been proposed, minimizing the requirement of physical resources by collecting data *remotely* (reducing or completely eliminating in-person clinical examinations) [7-10]. In 2015, the NIH funded a national resource to *mobilize research* by creating an infrastructure for conducting research using mHealth technology and has recently initiated the All of Us Research Program (formerly called the Precision Medicine Initiative) [11]. The All of Us program is a large, national study, with the goal of recruiting 1 million participants, which differs from other national cohorts such as the United Kingdom Biobank Study [12], by allowing for electronic (remote) enrollment. Successful recruitment in previous e-Cohort studies such as Health eHeart (HeH) Study and MyHeart Counts, which do not require on-site visits [13,14], have paved the way for new, large e-Cohorts such as All of Us.

The e-Cohort approach may provide a cost-effective methodology to remotely collect population-level data outside of standard research clinic settings, using mHealth devices and internet-based questionnaires [7-10], but may introduce substantial selection bias beyond that of typical research studies [13,15]. Investigators from HeH reported that HeH participants are more likely to be female, white/non-Hispanic, college-educated, nonsmokers, in excellent general health, but are also more likely to have cardiovascular disease and risk factors, compared with a national research study with more traditional recruitment practices [13]. Moreover, the level of technical support that may be required by participants for mHealth device data collection is unclear, especially with regard to middle-aged and older adults who may have less familiarity and require more support with mHealth technology [16]. Finally, despite several theoretical advantages of merging these newer remote studies (lacking on-site visits) with established conventional cohorts, this practice has not yet been carefully studied [6].

### Objectives

We conducted a 5-month pilot study in the well-characterized Framingham Heart Study (FHS) cohort to test the feasibility of incorporating mHealth technology in a long-standing epidemiologic cohort study using remote versus in-person device set up. Our approach to pilot test and scale up the use of mHealth technology and electronic surveys (e-surveys) within FHS [17] leveraged the committed study participants and infrastructure of FHS. For the pilot study, we partnered with the HeH Study, which had an established protocol and infrastructure for collecting mHealth data.

The main purpose of our FHS-HeH pilot study was to assess whether remote mHealth data collection supported by email was equivalent to a strategy that involved in-person support *on-site* at the FHS Research Center by measuring the rates of mHealth device set up and continued use over the 5-month study. In addition to testing the feasibility and optimal data collection strategy, we also assessed the clinical characteristics of enrolled versus declined participants, completion rates of internet-based self-report data, and study design acceptability among participants.

## Methods

### Study Design

The FHS began enrolling participants for the Original cohort in 1948 [18]. In 1971, the offspring of the Original cohort and the spouses of these offspring were enrolled in the Offspring study [19]. In 1994 and 2002, ethnic/racial minority Omni cohorts were recruited to increase the diversity represented in FHS to better reflect the contemporary diversity of the town of Framingham, Massachusetts. In addition, in 2002, Third Generation participants were recruited from a sample of individuals that had at least 1 parent in the Offspring cohort [20]. These participants have been followed at 2- to 8-year intervals in the subsequent years and the study is ongoing. The most recent Offspring examination (including Omni cohort 1) occurred between 2011 and 2014 and the last Third Generation (including Omni cohort 2) examination was conducted during 2008 to 2011. Previous FHS examinations primarily used phone calls to recruit participants to return to the FHS Research Center.

**Figure 1:**
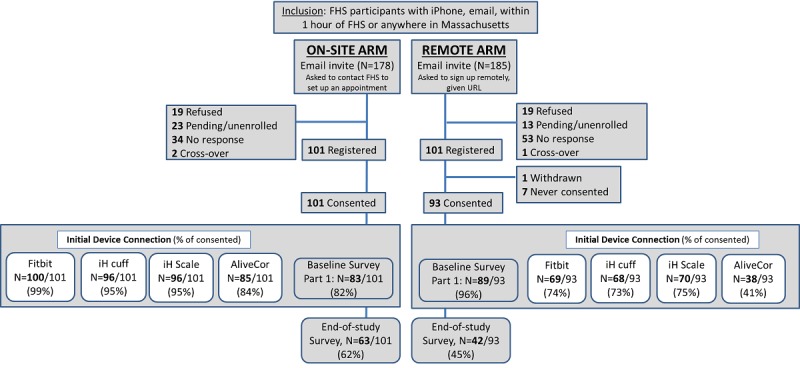
Flow chart of recruitment and initial device connection for the Framingham Heart Study–Health eHeart pilot study. Pending/unenrolled participants responded to the initial email invitation, but they did not respond to further communications. FHS: Framingham Heart Study; iH: iHealth; AliveCor: electrocardiogram device.

FHS Offspring, Third Generation, and Omni participants [19,20] who had an email address, lived within a 1-hour drive of the FHS Research Center and owned an iPhone [21] were eligible for participation in this investigation. The iPhone requirement was included as, at the time, not all devices were supported by Android. A previous report from FHS, Framingham Digital Connectedness Survey, permitted us to identify participants reporting iPhone ownership and internet use for recruitment purposes [21]. During the recruitment (May-October 2015), 363 participants were sent an email invitation Figure 1. Our goal was to recruit 100 participants in each of the study arms (*remote* vs *on-site* support) and to sample at least 20% older participants (age ≥65 years). Our study protocol followed Zelen design [22], in which participants were randomized to one of the following 2 groups before invitations were sent and consent was obtained:

- Remote support: Participants randomized to the remote support group received an email invitation with an explanation about the FHS-HeH pilot study and a URL they could follow to learn more and register for the study (first figure, Multimedia Appendix 1). For those who did not register within 1 week of the initial email, a second email was sent. After a second week of no response, a phone call was placed to their home. No more than 3 phone calls were placed to any individual for recruitment purposes.

- On-site support: Participants randomized to the on-site support group were contacted by the same email/phone call protocol to register for the study and set up a study visit (second figure, Multimedia Appendix 1). Trained FHS staff members assisted the participants in-person to register with the FHS-HeH pilot study, sign the Web-based consent, and connect the devices to their iPhones and the study website. If requested, participants were able to return to the FHS Center if they required additional in-person support.

After the study termination (March 2016), all participants were emailed an *end-of-study survey*, through an internet link, to assess the participant burden and the overall FHS-HeH experience. The survey went out after 98% of the participants had completed the 5-month study (4 participants had not yet completed 5 months). The FHS-HeH study was approved by the Institutional Review Board (IRB) at the University of California, San Francisco, and the participants provided written informed consent. The Boston University Medical Center had an approved IRB authorization agreement.

### Covariates

The following demographic information was collected from the most recent FHS examination attended: age, sex, body mass index (BMI), physical activity index [23], history of smoking (defined as former or current smokers, having at least 1 cigarette per day in the past year), hyperlipidemia (total cholesterol ≥200 mg/dL or being on lipid treatment), education, diabetes mellitus (defined as fasting glucose ≥126 mg/dL or treatment with hypoglycemic agent or insulin), hypertension (defined as systolic BP ≥140 mm Hg or diastolic BP ≥90 mm Hg or being on treatment), atrial fibrillation, and cardiovascular disease (includes myocardial infarction, coronary insufficiency, atherothrombotic brain infarct, transient ischemic attack, intermittent claudication, and heart failure). Participants with missing demographic data (detailed in the Results section) either did not attend their last FHS examination cycle or did not complete that part of the examination. Participants with missing covariate data were included in all tables.

### Statistical Analysis

Demographic information was reported as mean (SD) for each study arm and for FHS participants who declined to participate in this investigation. Study adherence was defined conservatively as simply taking 1 measurement each month to get a broad assessment of continued device use. Study adherence and survey responses were compared between the 2 study arms in the total study sample by calculating the mean percent differences and 95% CIs. All statistical analyses were performed by using SAS, version 8 (SAS Institute Inc). Significant differences were reported at the *P*<.05 level.

## Results

### Study Enrollment

Of the 363 participants invited, 87 participants did not respond to the initial recruitment efforts, 38 declined to participate, and 36 communicated an intent to participate but did not follow through with enrollment (Figure 1). There were 101 participants who completed enrollment in each of the randomized study arms (n=202 total). Owing to the 2 early withdrawals (1 withdrawal in each study arm), additional participants were allowed to enroll to replace these withdrawals. In the *on-site* arm, there was a study technician available to answer questions and we observed 100% completion of the consent process. In contrast, individuals in the *remote* arm were emailed a link to initiate the consent process; only 93/101 (92%) completed the consent. In total, 82 participants responded to the invitation but did not complete the consent (38 participants declined, 36 were pending/not enrolled, and 8 enrolled but did not complete consent). Consenting participants were more likely to be women, tended to be younger, were less likely to smoke or have diabetes mellitus, and were more likely to have attended at least some college (Table 1). The rates of missing demographic data from Table 1 were low (BMI, missing [m]=11; physical activity index, m=13; history of smoking, m=3; hyperlipidemia, m=11; education, m=5; diabetes mellitus, m=15; and hypertension, m=11). Missing data were because of either missing the most recent FHS examination or missing the questionnaire/biomarker data at the most recent examination. None of the participants missing diabetes mellitus data had a diagnosis of diabetes mellitus on FHS examinations that occurred before the most recent FHS examination.

**Table 1:** Demographic information from study participants collected at their last Framingham Heart Study examination.

Demographics		Consented to study (n=194)		Responded to invitation, but not consented^a^ (n=82)	*P* value for difference between consented and not consented^b^
		Randomized to on-site arm (n=101)	Randomized to remote arm (n=93)		
Age (years), mean (SD)		55 (11)	53 (10)	58 (12)	.009
Women, n (%)		60 (59)	57 (61)	38 (46)	.04
**Cohort, n (%)**					
	Offspring	19 (19)	12 (13)	30 (37)	—^c^
	Third Generation	76 (75)	75 (81)	49 (60)	—
	Omni 1	2 (2)	—	—	—
	Omni 2	4 (4)	6 (6)	3 (4)	—
**Education, n (%)**					
	Less than high school	—	—	—	—
	High school	6 (6)	3 (3)	14 (17)	—
	Some college	10 (10)	19 (20)	17 (21)	—
	College and higher	85 (84)	71 (76)	51 (62)	—
Body mass index (kg/m^2^), mean (SD)		27 (5)	29 (6)	28 (6)	.48
Physical Activity Index, mean (SD)		35 (5)	35 (7)	36 (5)	.26
History of smoking, n (%)		15 (15)	21 (23)	29 (35)	.002
Hyperlipidemia, n (%)		47 (46)	47 (53)	40 (50)	.99
Diabetes mellitus, n (%)		5 (5)	2 (2)	8 (10)	.09
Hypertension, n (%)		20 (20)	22 (25)	19 (24)	.87
Cardiovascular disease, n (%)		2 (2)	7 (8)	4 (5)	.99
Atrial fibrillation, n (%)		2 (2)	1 (1)	1 (1)	.99

^a^The *not consented* column includes 38 participants who declined, 36 pending/not enrolled, and 8 enrolled but did not complete consent.

^b^*P* values were not calculated for differences in cohort and education because of low numbers in some groups.

^c^Not applicable.

Importantly, recruitment of the older adults (age ≥65 years) for this e-Cohort study was less efficient (50% of individuals consented, 27 out of the 54 individuals who responded to the email invitation to participate) compared with the recruitment of adults aged <65 years (75% consented, 167 out of the 222 individuals who responded to the email invitation), as calculated from Table 1 and the first table of Multimedia Appendix 1. Older adults choosing to participate in our study had completed more education (100% completing at least some college) than those choosing not to participate, of which 26% (n=7/27) had not continued on to college after high school.

### Device Use

In the *on-site* arm, 99% of the consenting participants (100/101) initially connected to the Fitbit device, 95% (96/101) to the iHealth BP cuff and scale, and 84% (85/101) to the AliveCor ECG. As for the *remote* arm, 74% of those that consented initially (69/93) connected to the Fitbit device, 73% (n=68/93) to the iHealth BP cuff, 75% (70/93) to the iHealth scale, and 41% (38/93) to the AliveCor ECG (Figure 1 and Table 2). The *on-site* arm had 20% to 43% more participants initially connected to the devices at baseline (mean percent difference was 25% [95% CI 17-35] for activity monitor, 22% [95% CI 12-32] for BP cuff, 20% [95% CI 10-30] for scale, and 43% [95% CI 30-55] for ECG).

**Figure 2:**
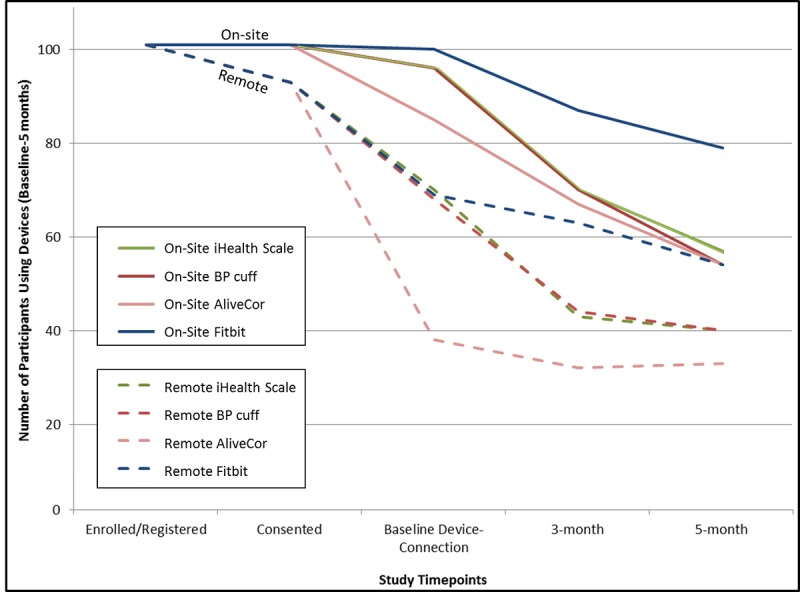
Number of participants using devices throughout the study from study enrollment through the 5-month follow-up period. BP: blood pressure.

After the initial connection, the proportion of participants that continued to use the devices declined consistently in both arms of the study (Table 3 and Figure 2). Although 4 study participants in the *on-site* arm did not have the opportunity to participate in the full 5-month study, removal of these participants in sensitivity analyses did not change the results considerably (second and third table of Multimedia Appendix 1).

**Table 2:** Primary analysis: Rate of device connection at baseline and continued use at 5 months.

Device	On-site (n=101), n (% consent)		Remote (n=93), n (% consent)		Difference in proportion of device connection rate between study arms	
	Baseline connection	Fifth month device use^a^	Baseline connection	Fifth month device use	Mean percent difference between study arms in baseline connection rate (95% CI)	Mean percent difference between study arms in fifth month device use rate (95% CI)
						
Fitbit device	100 (99)	79 (78)	69 (74)	54 (58)	25 (17 to 35)	20 (7 to 33)
iHealth blood pressure cuff	96 (95)	54 (53)	68 (73)	40 (43)	22 (12 to 32)	10 (−4 to 24)
iHealth scale	96 (95)	57 (56)	70 (75)	40 (43)	20 (10 to 30)	13 (−1 to 27)
AliveCor	85 (84)	54 (53)	38 (41)	33 (35)	43 (30 to 55)	18 (4 to 31)

^a^A total of 4 participants in the *on-site* arm did not have the opportunity to participate for the full 5 months owing to study termination.

**Table 3:** Secondary analysis: Continued use of devices for participants who were initially able to connect to the devices during the first month. The n (%) values are given with regard to baseline device connection.

Device	On-site (N=101)			Remote (N=93)			Difference in proportion of continued device use between study arms	
	Baseline connection, n	Third month device use, n (% baseline)	Fifth month device use, n (% baseline)^a^	Baseline connection, n	Third month device use, n (% baseline)	Fifth month device use, n (% baseline)	Mean percentage difference between study arms in baseline connection rate (95% CI)	Mean percentage difference between study arms in fifth month device use rate (95% CI)
								
Fitbit device	100	87 (86)	79 (79)	69	63 (91)	54 (78)	−4 (−14 to 6)	1 (−12 to 14)
iHealth blood pressure cuff	96	70 (69)	54 (56)	68	44 (65)	40 (59)	8 (−6 to 23)	−3 (−18 to 13)
iHealth scale	96	70 (69)	57 (59)	70	43 (61)	40 (57)	11 (−3 to 26)	2 (−13 to 17)
AliveCor	85	67 (66)	54 (64)	38	32 (84)	33 (87)	−5 (−9 to 11)	−23 (−37 to −6)

^a^A total of 4 participants in the *on-site* arm did not have the opportunity to participate for the full 5 months owing to study termination.

### Survey Data

All consenting participants were sent links to participate in the 2 internet-based surveys: a baseline core survey and an *end-of-study survey* after the study termination. The baseline core survey comprised 34 separate parts assessing self-reported health outcomes that could be completed in any order and was well attended by participants in both arms. The first survey was completed by 83 (82%) participants from the *on-site* arm and 89 (96%) participants from the *remote* arm (Figure 1.) After the study completion, all participants were sent an end-of-study survey, of which only 63% of the *on-site* arm and 45% of the *remote* arm participated (fourth table, Multimedia Appendix 1). Overall, the participants endorsed positive statements about their study participation. At least 95% of the participants in both study arms agreed to the statement, “I would participate in this type of study again in the future.” Over 85% of the participants in the *on-site* arm agreed with almost all the survey questions (as demonstrated by the shaded boxes in the fourth table of Multimedia Appendix 1), whereas there was slightly lower agreement for the *remote* arm.

## Discussion

### Principal Findings

Our FHS-HeH pilot study was conducted in collaboration with the HeH Study to test feasibility of mHealth and digital data collection in FHS participants using remote versus in-person support for device set up and use over a 5-month period. Participants in our *on-site* study arm had the opportunity to visit the FHS Research Center for consent and mobile device set up. We observed that the *on-site* participants were more likely to consent and had better success with initial device connection and use compared with the individuals who received only *remote* support by phone or email. However, once connected to the devices, the rates of continued device use were similar in both groups. Our findings suggest that it is possible to maximize participation by leveraging in-person support for e-Cohort studies. Furthermore, we observed reasonable adherence with mHealth technology by older adults.

In both study arms combined, almost 79% of the participants who successfully initialized the Fitbit device at the beginning of the study continued to use the device for the full 5-month study, representing 69% of the total sample of consenting study participants. We defined *continued use* very conservatively, as 1 measurement per month, to get a broad assessment of continued device use. Preliminary data from a new FHS initiative separate from FHS-HeH, called *eFHS*, reported that 76% (306 of 402 participants given an Apple Watch device) wore the device at least weekly over 3 months and received reminder messages if no data were sent for 14 days [17].

In 2 other studies that recruited participants using *snowball* (social network/internet-based) sampling strategies specifically to enroll participants into e-Cohort studies, surprisingly, the frequency of device use did not appear to be more successful, and may have even been lower, than in FHS-HeH or eFHS which enrolled from within the ongoing FHS cohort [14,24]. In the MyHeart Counts study, investigators reported that 47% of their >48,000 consented study participants completed just 2 consecutive days of fitness monitor data as measured by a smartphone app in the first week and adherence only declined from there [14]. In the mPower substudy of HeH, a 6-month smartphone-based study, 87% of 9520 study participants completed at least one task on the smartphone app after consenting to the study, but only 9% contributed data on ≥5 separate days, confirming that consistency in device or app use is one of the major challenges of this type of research [24]. Physical activity intervention studies provide additional comparative data, with considerable drop-off in device use over the short term (3-6 months) and over longer periods (6 months to 1 year), especially after the participant incentives are terminated [25-27]. Unfortunately, owing to our study termination after at least 5-months follow-up, we are unable to test whether there would be an effect of device setup strategy (on-site versus remote) on longer follow-up of continued device use. It is also unclear what type of communication, support, or incentives might maximize adherence with mHealth devices. In our study, participants were only sent reminders to sync their devices, briefly, midstudy. Our study was not designed to assess whether these reminders affected device use. However, there is a burgeoning field of study testing communication methods/strategies to increase and sustain health behavior [28-31]. Messaging may need to be tailored to participants based on the current adherence, and investigators should be cautious that the language does not infer that data are not received, unless that is the message meant to be communicated.

Overall use of the BP cuff, scale, and AliveCor ECG were somewhat lower than the continued use of the Fitbit device in our FHS-HeH *on-site* arm, but generally, once connected, the use was similar for both the study arms. Across both arms, 56% to 59% of the participants who successfully connected the BP cuff or scale at baseline, continued to use it through the 5-month study duration. For comparison, in a meta-analysis, rates of adherence to self-monitoring BP in hypertensive patients participating in an intervention to lower BP varied widely by study, but true comparison with our study is difficult as most of our FHS-HeH participants were not hypertensive [32]. In addition, most studies from the meta-analysis used traditional nonconnected BP devices, instead of mHealth devices with smartphone apps.

Device connection to the AliveCor ECG device was lower than other devices. Our technical staff reported that the AliveCor was typically the last device they connected during the in-person visit. Another contributing factor could be the more complex instructions for setting up the AliveCor device, including multiple steps in which the participants were required to log in to their email. Other than these reasons, it is unclear why the connection rates were much lower in the remote arm (41%) compared with the on-site arm (84%).

In contrast to the diminishing rates of the BP cuff or scale use over time, the rate of continued adherence for those that were initially able to connect to the AliveCor ECG remained relatively high at 5 months (especially in the remote arm, 87%). However, enthusiasm about apparently high AliveCor adherence should be tempered by the fact that only a small number of participants connected to this device at baseline. Thus, participants who successfully connected to the AliveCor at baseline may differ from those who connected to other devices. We hypothesize that AliveCor users may be more interested in their health, more motivated study participants, and/or more technologically savvy. However, one limitation to our study is that we did not measure the reasons for differences in device use, so we are not able to determine the facilitators and barriers to the use of specific devices [13,14,16,33,34].

### Internet-Based Survey Data Can Be Successfully Administered Via Different Strategies

In addition to answering important questions about device connection and use, our study was able to assess the rate of internet-based survey initiation using our 2 study arms. Until recently, the FHS has conducted most questionnaires in-person and only administered short health history updates in the interim between examinations by phone or using traditional mail via the postal service. Although consent and device connection appeared more successful in the *on-site* arm of the study, the participation in the baseline core survey was higher in the remote arm (82% vs 96% in the *on-site* and *remote* arms, respectively). Therefore, in-person contact may not be an important part of a study designed only to perform surveys with participants. Instead, higher survey participation rates in the *remote* arm may be reflective of the lower burden imposed initially in the *remote* arm before devices were shipped. These results provide some evidence that internet-based surveys may be effective means to conduct a health history questionnaire in FHS participants.

Other e-Cohort studies have had variable success with participant engagement in e-surveys, which may depend on the timing and strategies used to present surveys to participants. In MyHeart Counts, 41% of the study participants completed a cardiovascular health survey, whereas 73% completed a physical activity survey, and only 17% provided race/ethnicity [14]. The HeH Study (with >210,000 participants) reported that 86% of participants completed at least one survey, but 37% provided complete survey data [13]. Another traditional cardiovascular epidemiology cohort, Coronary Artery Risk Development in Young Adults has also explored the electronic administration of surveys through the internet (eCARDIA), reporting 52% survey completion [35]. On the basis of the results from these studies, it may be important to prioritize survey administration in e-Cohort studies to ensure that the most important surveys have strong adherence.

In contrast to the high participation rates in the baseline core survey, the end-of-study survey was not completed as frequently (62% vs 45% of *on-site* and *remote* participants, respectively). Study design and communication with participants are not only important for the baseline connection and use of the device, but also for good adherence to device use at follow-up. These considerations are especially important for longitudinal studies that continue to engage participants over a long follow-up period as poor communication and frustration from participants may impact future participation. On the basis of data from approximately half of the study participants who provided feedback, approximately 96% of the participants said that they would participate in this type of study again, regardless of the study arm. Although participation bias influences our ability to interpret results from the end-of-study survey, it does appear that the *on-site* participants responded more favorably overall.

### Strengths, Bias, and Limitations of Our Study Design

The strengths of our study lie in our study design, which leveraged infrastructure and the strengths of FHS and HeH, including a recruitment sample of committed study participants across middle and older age. Our design not only enabled the examination of different methodologies for incorporating consumer-facing mHealth technology into an epidemiological study, but may also provide insight for other study designs, including clinical trials.

Important limitations to consider include the limited exploration of participation bias by demographic factors other than age. The study was small, and we had limited power to examine subgroup findings. The FHS primarily comprises white individuals residing in New England; therefore, we were unable to analyze how the study design influenced participation by racial/ethnic group or region. Certain demographic groups may be more unlikely to be eligible for participation in mHealth studies, such as those that do not have a smartphone [21]. In our FHS *Digital Connectedness Survey*, administered during 2014 to 2015, we reported that smartphone users in FHS were younger, more highly educated, with less cardiovascular risk factors than individuals without a smartphone [21]. However, even among the participants who were eligible for our study (ie, had an iPhone and email address), those who agreed to participate were more likely to have attended at least some college (95% vs 82% among participants who were eligible but declined to participate) and were less likely to be smokers. Both trends are similar to what was seen in other e-Cohort studies [35,36], including the preliminary HeH recruitment analysis in which participants were less likely to smoke and were more likely to be women, had higher educational attainment, reported excellent general health, and were likely to be white (rather than black, Hispanic, or Asian) when compared with the traditional National Health and Nutrition Examination Survey study design [13]. Although issues of generalizability plague all epidemiological studies, it may be a particular concern in e-Cohort studies.

Previous studies in minority communities in the United States cited concerns and misconceptions by the participants in mHealth studies, such as the type of information that would be tracked by mobile technology, legal risks that might be introduced through participation, a lack of familiarity with certain devices, and unwanted attention from others when wearing or using devices in public [37,38]. These concerns can impact both study participation and adherence and may require cultural sensitivity (or age/generational sensitivity), creativity, and patience from the study team. The study team must weigh cost-effectiveness of potential adaptations, with limiting selection bias and maximizing the equity in research across diverse populations [37,38]. We did not analyze the cost between the study arms, so we are not able to compare the differences in our study. It is possible that personal communications with knowledgeable study coordinators and the research team may help to overcome some of the barriers mentioned above. The introduction of mHealth technology raised some concerns even in FHS participants who are familiar with research studies. We observed a barrier to consent that was somewhat overcome through the *on-site* study design, in which participants spoke with study coordinators who could explain the study, answer questions, and provide in-person support for setting up the mHealth devices. Future studies should assess whether other forms of participant engagement, such as text messaging, will influence mobile device use and study adherence.

We also acknowledge the conservative measure of study adherence (device use once per month) as we were most interested in assessing the overall adherence as a primary study aim. In future studies, it will also be important to understand the barriers preventing study adherence and to investigate the factors contributing to the frequency of use and how to improve these metrics. We acknowledge that providing 4 devices might have been burdensome for some participants, especially as participants needed to visit 3 different consumer-facing websites/apps to create accounts for each device (iHealth, Fitbit, and AliveCor) to connect the devices to the HeH platform, adding complexity to the initial user experience. Using 1 single app to connect multiple devices may improve connection for participants, especially for participants connecting remotely. Another key future step will be testing different methods of supporting and engaging participants, including assessing how participants engage with the website/apps using Web analytics tools. Providing in-person support, as we showed in our FHS-HeH pilot study, has the potential to increase study efficiency and may also minimize participation bias.

### Conclusions

Our feasibility study demonstrated that offering *on-site* support for studies involving mHealth technology maximizes participation and initial rates of device use, compared with offering only *remote* support. However, once connected, drop-off rates were similar in both groups. Future studies may find it to be cost-effective to provide in-person support for studies involving mHealth technology for middle-aged and older populations.

## Appendix

Multimedia Appendix 1 Supplemental material containing the participant recruitment emails and tables including demographics of participants at least 65 years of age, sensitivity analyses, and "end of study" survey responses.

Multimedia Appendix 2 CONSORT‐EHEALTH checklist (V 1.6.1).

## Abbreviations

BMI: body mass index
BP: blood pressure
ECG: electrocardiogram
e-Cohort: electronic cohort
e-survey: electronic survey
FHS: Framingham Heart Study
HeH: Health eHeart
HHS: Health and Human Services
IRB: Institutional Review Board
mHealth: mobile health
NHLBI: National Heart, Lung, and Blood Institute
NIH: National Institutes of Health
