# Supplementary material for: Comparison of On-Site Versus Remote Mobile Device Support in the Framingham Heart Study Using the Health eHeart Study for Digital Follow-up: Randomized Pilot Study Set Within an Observational Study Design
Source: JMIR Mhealth Uhealth. 2019 Sep 30;7(9):e13238. doi: 10.2196/13238 (PMC6792023; doi:10.2196/13238)
Supplement: Multimedia Appendix 1 [file mhealth_v7i9e13238_app1.pdf]

SUPPLEMENTAL MATERIAL

Supplemental Figure 1A. Email recruitment for the "On-Site" arm, with the paragraph deviating from the "Remote" arm recruitment letter boxed in red.

Dear «PreferredName»,

Thank you for your ongoing participation in the Framingham Heart Study (FHS).

We are developing and testing a new way to collect data in the FHS using the internet, smartphones, and wearable devices. The purpose of this study is to determine whether electronic data collection is possible in FHS. If possible, this would help pave the way for a larger study in all interested FHS participants.

We have selected you because you previously told us that you have an active email account, an iPhone, and live within one-hour of FHS. We have partnered with The Health eHeart Study from the University of California, San Francisco for this pilot study.

If you choose to participate in this pilot, you will be asked to:

1. Register with the FHS-Health eHeart Pilot Study, by using your email address as a login and creating a password.
2. Sign our online consent.
3. Complete a set of FHS-Health eHeart Pilot Study surveys online.
4. Connect up to four devices (that we will provide to you) to your iPhone – a Fitbit activity monitor, an iHealth blood pressure cuff, an iHealth scale and an AliveCor ECG iPhone case.
5. Download a smartphone app (Ginger.io).

Each of these will be explained in detail during the consenting process and you will be able opt out of any part. We anticipate that this study will last up to 12 months. Participation in this study does not impact your participation in the FHS.

To help you with getting set up for this study, we invite you to come in to the FHS. At this visit, we will walk you through the sign up and consent process, give you your devices, and help you set them up. Please call or email Emily Manders [redacted] to schedule an appointment.

Joining the FHS – Health eHeart Pilot Study is voluntary. If you decide not to participate, your choice will not affect your relationship with the FHS in any way. If you have questions we would be happy to answer them - please call [redacted] or email us [redacted]

Sincerely,

Caroline S. Fox MD MPH
Joanne M. Murabito MD MSc
Emelia J. Benjamin MD MSc

Note: The Ginger.io app did not end of being a component of the final FHS-HeH pilot study design

Supplemental Figure 1B. The paragraph from the “Remote” arm recruitment email that differs from the “On-site” recruitment email (replaced red box in Supplemental Figure 1A)

You can join the Framingham Heart Study – Health eHeart Pilot Study today. If you want to learn more or sign up for the study, please visit the study website by clicking the link below (or copy/pasting it into your browser):

PARTICIPANT’S UNIQUE URL

You will be taken to a special login page created just for FHS participants. To sign up for the study, click on the “Learn More” button and follow the on-screen instructions.

Supplemental Table 1. Demographic information from study participants collected at their last Framingham Heart Study examination in mean ± standard deviation or as percent (%) of each study arm, restricting to participants that were **≥65 years old**

Demographics	Consented to study N=27		Responded to Invitation, but Not Consented n=27	p-value for difference between consented and not consented
	Randomized to "On-Site" Arm N=17	Randomized to "Remote" Arm N=10		
Age, y	71 ± 5	70 ± 4	71 ± 6	0.86
Women (%)	47%	50%	52%	1.0
Cohort				
Offspring (%)	88%	80%	85%	
Third Generation (%)	12%	20%	15%	
Omni 1 (%)	--	--	--	
Omni 2 (%)	--	--	--	
Education				
Less than High School (%)	--	--	--	
High School (%)	--	--	26%	
Some College (%)	6%	20%	26%	
College and Higher (%)	94%	80%	48%	
BMI, kg/m^2	27 ± 4	31 ± 6	28 ± 4	0.41
Physical Activity Index	35 ± 4	32 ± 2	36 ± 6	0.14
History of Smoking (%)	35%	70%	52%	1.0
Hyperlipidemia (%)	81%	90%	63%	0.12
Diabetes Mellitus (%)	19%	10%	15%	1.0
Hypertension (%)	38%	80%	33%	0.17
Cardiovascular Disease (%)	12%	20%	11%	1.0
Atrial Fibrillation (%)	12%	10%	4%	0.61
Data depicted as mean ± standard deviation or as % of each study arm				

Supplemental Table 2. Sensitivity of the primary analysis: Rate of devices connection at baseline and continued use at 5 months, n (% of those consenting to the study, excluding 4 participants in the *on-site* arm who did not have the opportunity to participate for the full 5 months due to study termination)

	On-Site n=97		Remote n=93		Difference of **proportion in device connection rate** between study arms	
	Baseline Connection	5th month device use	Baseline Connection	5th month device use	**Mean % Difference** between study arms **in Baseline Connection** rate, 95% CI	**Mean % Difference** between study arms **in 5th month device use** rate, 95% CI
	n (% consent)	n (% consent)	n (% consent)	n (% consent)		
Fitbit	96 (99%)	77 (79%)	69 (74%)	54 (58%)	25% (16,35)	21% (8,34)
iHealth BP Cuff	92 (95%)	52 (54%)	68 (73%)	40 (43%)	22% (12,32)	11% (-4,24)
iHealth Scale	92 (95%)	54 (56%)	70 (75%)	40 (43%)	20% (10,30)	13% (-2,26)
AliveCor	82 (85%)	53 (55%)	38 (41%)	33 (35%)	44% (31,55)	19% (5,33)

Supplemental Table 3. Sensitivity of the secondary analysis: Continued use of devices for participants who were initially able to connect to devices during the 1st month, n (% of baseline device connection, excluding 4 participants in the *on-site* arm who did not have the opportunity to participate for the full 5 months due to study termination)

	On-Site n=97			Remote n=93			Difference of proportion in continued device use between study arms	
	Baseline Connection (1st month)	3rd month device use	5th month device use	Baseline Connection (1st month)	3rd month device use	5th month device use	**Mean % Difference** between study arms **in Baseline Connection** rate, 95% CI	**Mean % Difference** between study arms **in 5th month device use** rate, 95% CI
	n	n (% baseline)	n (% baseline)	n	n (% baseline)	n (% baseline)		
Fitbit	96	84 (88%)	77 (80%)	69	63 (91%)	54 (78%)	-4% (-13,7)	2% (-10,15)
iHealth BP Cuff	92	66 (72%)	52 (57%)	68	44 (65%)	40 (59%)	7% (-7,22)	-2% (-17,13)
iHealth Scale	92	66 (72%)	54 (59%)	70	43 (61%)	40 (57%)	10% (-4,25)	2% (-14,17)
AliveCor	82	64 (78%)	53 (65%)	38	32 (84%)	33 (87%)	-6% (-20,10)	-22% (-36,-5)

Supplemental Table 4. Percent of end of study survey responders that answered in agreement to questions (responding 1="strongly agree," or 2="agree", on a scale of 1-5, [5=strongly disagree])

N (%) responding with agreement (either "1" or "2") to end of study survey statements	On-Site (n=63 of 101 that consented, 62%)	Remote (n=42 of 93 that consented, 45%)
"I would participate in this type of study again in the future"	61 (97%)	40 (95%)
"It was easy to follow the written instructions on how to set up device(s)"	52 (83%)	31 (76%)[a]
"I was able to get the help I needed for setting up device(s)"	57 (90%)	39 (95%)[a]
"It was easy to set up the device(s) with my phone and/or computer"	55 (87%)	33 (80%)[a]
"It was easy to link up device(s) with the Health eHeart website"	54 (86%)	30 (73%)[a]
"It was easy using the device(s)"	55 (87%)	33 (80%)[a]

[a]1 participant in the remote group did not respond to most of the survey questions. For these responses, percent was calculated out of n=41 responses.

Those with >85% agreement (either "agreed" or "strongly agreed") are shaded in grey
